# Construction and Application of Bridge Expansion and Contraction Installation Replacement Decision System Based on the Analytic Hierarchy Process

**DOI:** 10.3390/ma13184177

**Published:** 2020-09-20

**Authors:** Minshui Huang, Zian Xu, Liang Li, Yongzhi Lei

**Affiliations:** Wuhan Institute of Technology, School of Civil Engineering and Architecture, Wuhan 430073, China; xuzian@wit.edu.cn (Z.X.); liliang1@citic.com (L.L.); mr_lei.yz@wit.edu.cn (Y.L.)

**Keywords:** bridge expansion and contraction installation (BECI), replacement method, functional index evaluation, life-cycle value assessment (LCVA), analytic hierarchy process (AHP), decision system

## Abstract

Bridge expansion and contraction installation (BECI) has proved to be an indispensable component of bridge structures due to its stability, comfort, and durability benefits. At present, conventional replacement technologies for modular-type, comb plate-type, and seamless-type BECIs are widely applied worldwide. However, it is unfortunate that there remains no systematic research on quantitative assessment approaches for evaluating the overall technical status and selecting optimal replacement methods for existing BECIs. Therefore, considering the installation performance according to functional index evaluations and the economic cost based on life-cycle value assessment (LCVA), a standardized quantitative assessment approach is proposed for optimal replacement method selection in this article. Simultaneously, the other new quantitative assessment method is developed for evaluating the overall technical status of BECIs, which provides a basis for the necessity of replacement. A BECI replacement decision system is constructed, and a corresponding case study illustrates that the proposed system based on the analytic hierarchy process (AHP) in this article proves to be reasonable and feasible. The results reveal that the selected replacement method with both a higher function coefficient and a lower economic coefficient can not only fulfil the performance requirements but also pursue a cost reduction, which leads to a considerable value increment. This system can effectively assist bridge managers in making appropriate operation and maintenance (O and M) decisions in actual engineering projects.

## 1. Introduction

With the rapid increase in technical obstacles caused by the damage of bridge expansion and contraction installations (BECIs), related economic burdens, traffic problems, and social arguments have attracted a great deal of attention. It is an urgent task to complete the replacement decision system for a BECI on a highway bridge with minimal negative impacts on installation performance and considerable achievements in resource conservation. According to the types of installation and the construction characteristics, the replacement technologies for BECIs can be generally divided into comb plate-type replacement, modular-type replacement, and seamless-type replacement [1].

The replacement of BECI appears a significant part in the bridge O and M field, where the decision and optimization of the O and M plan are highly prized. In many studies, the framework of the decision-making system is usually divided into the network layer and the project layer; the former is used to ensure the optimal project choice, and the latter guarantees that resources are efficiently allocated [2]. Referring to the research of Cao et al. [3], a decision-making system for pavement recycling is established based on the analytic hierarchy process (AHP) in such a framework. Similarly, many studies based on AHP and fuzzy AHP have been performed on the problems at the network layer [4,5,6,7,8,9]. As a potential decision-making (DM) method, AHP is often used to analyze and solve engineering problems, such as contractor prequalification [10], safety risk assessment [11], selection of the right contractor, and identification and evaluation of the critical success indices for construction projects [12,13,14]. Compared with the other common multi-criterion decision-making (MCDM) methods, such as the analytic network process (ANP), preference ranking organization method for enrichment evaluations (PROMETHEE), simple additive weighting (SAW), and the technique for order of preference by similarity to ideal solution (TOPSIS), AHP has higher applicability and effectiveness [15]. In addition, in order to overcome DM problems in civil engineering projects (CEPs), the AHP and fuzzy technique for order of preference by similarity to ideal solution (Fuzzy TOPSIS) is introduced [16], and the two techniques have the facility to be integrated and combined in a new module to support most of the decisions required in CEPs. At the same time, the fuzzy and AHP methods have been used in public-private partnership (PPP) projects for risk and traffic safety assessment of highways [17,18].

As a part of the BECI replacement decision system, the selection of the replacement method should comprehensively integrate the design requirements, construction requirements, management requirements, and the scope of the application when carrying out the maintenance plan at the network layer. However, an optimal BECI replacement should not only meet performance requirements but also pursue resource conservation and cost reduction. The method of life-cycle value assessment (LCVA) is often utilized to minimize potential impacts on cost consumption in the engineering field. One of the most important objectives of contract management is to allocate and use the limited resources to balance lifetime reliability and the whole life-cycle cost in an optimal manner [19]. LCVA is also implemented in contraction life-cycle management and a life-cycle analysis model is proposed to minimize the impact of buildings and dwelling home on environment [20,21]. Related studies have been conducted on concrete bridge design, deck applications, and retrofit assessment successfully [22,23,24]. In addition, value engineering (VE) is widely applied in the selection of optimal choice when carrying out comprehensive evaluations. VE has become an integral part of many construction projects that have sought a way to increase the value of projects [25,26], while meeting or exceeding the required function of a facility at a minimum life-cycle cost [27], and the value coefficient is expressed in terms of the function and cost: it is the ratio of the functional coefficient to the cost coefficient [28].

Many studies have been conducted on decision-making and plan optimization, whose achievements play a significant guiding role in later study and production. However, to date, there remains no developed system which can be utilized in replacement decisions for BECI. Meanwhile, the evaluation of the overall technical status for BECIs urgently need a quantitative assessment approach and, thus, motivates this article.

Based on the existing research results, a new quantitative assessment approach is proposed for evaluating the overall technical status of BECIs, which provides a basis for the necessity of replacement. Meanwhile, a quantitative assessment approach for selecting a replacement method is developed and a calculation model is established for economic evaluation. Consequently, an optimal replacement method is selected by the decision system to guide later bridge managers, and it also promotes the application of BECI replacement technologies in bridge O and M.

## 2. Objective and Methodology

**Figure 1:**
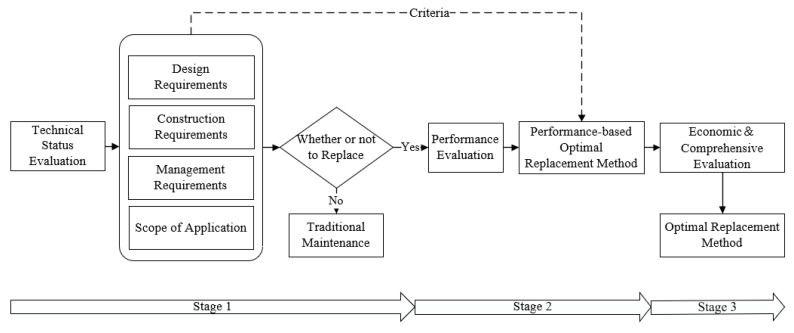
Decision-making process of the replacement decision system.

The optimal BECI replacement method selected by the constructed system should achieve the goals of two aspects, pursuing considerable installation performance condition and minimizing replacement cost consumption. As a premise to meet these requirements, the evaluation of BECI overall technical states should be conducted. Referring to the results of this evaluation, the necessity of replacement will be judged. If replacement should be performed, a performance evaluation based on AHP will be conducted; otherwise, traditional maintenance methods will be carried out instead. In particular, by regarding design requirements, construction requirements, management requirements, and the scope of the application as criteria, AHP can be efficiently utilized to rank the candidate replacement methods and select the performance-based optimal one, which is capable to ensure an excellent performance condition. Subsequently, economic evaluation and related comprehensive evaluation will follow, which eventually lead to the optimal replacement method that achieves both performance and economic goals. The decision-making process of the replacement decision system is shown in Figure 1.

Design requirements, construction requirements, management requirements, and the scope of the application provide a robust basis for the selection of a performance-based optimal replacement method. However, the more factors that are taken into account, the less accurate quantitative assessment approaches appear. As a consequence, in order to simplify and refine this method, the scope of the application, which mainly refers to the amount of expansion and contraction, is not considered in this research. The preselection of candidate BECI replacement methods according to the experience of decision-makers will be feasibly carried out instead. 

## 3. Design of the Replacement Decision System

The design process can be divided into three main phases based on the objectives of the established selection decision system and the performance and economic assessment methods.

Stage 1: Decision of replacement plan. Evaluation of the overall technical status of the BECI is performed, and whether or not to replace is determined.

Stage 2: Selection of replacement method according to AHP. Based on the design requirements, construction requirements, management requirements, and scope of application of the BECIs, the performance-based optimal replacement method is selected via AHP.

Stage 3: Economic evaluation of replacement method. Based on the conditions of initial cost, operation and maintenance cost, vehicle operating cost, traffic delay cost, accident cost, and life-cycle maintenance cost of each replacement method, a mathematical model with strong applicability is established for economic evaluation.

After the above three stages, a further comprehensive evaluation can be carried out to make the optimal replacement plan with excellent performance and economic benefits.

### 3.1. Decision of the Replacement Plan

**Figure 2:**
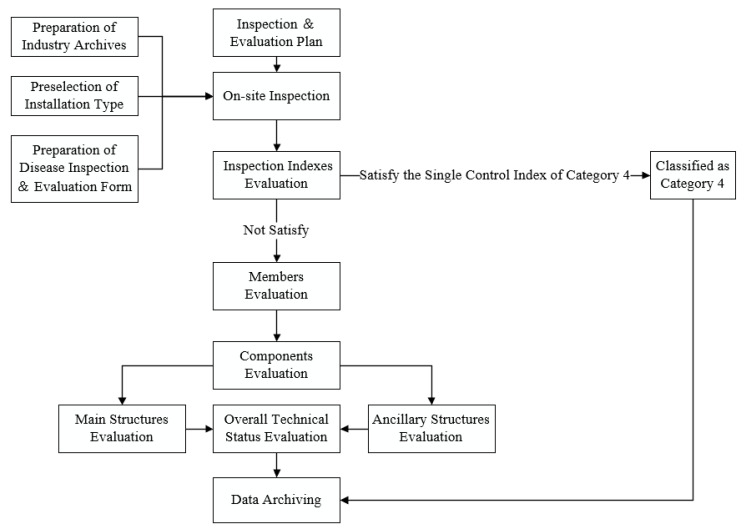
Decision tree of the BECI replacement plan.

In the process of this decision-making system, the overall technical status of the installation should be evaluated first. The decision tree of the BECI based on the above standards is shown in Figure 2.

The method of combining an itemized classification with a single control technical index is developed to classify the evaluation. The technical condition evaluation of the BECI, which combines the hierarchical comprehensive evaluation with the category 4 single control index of the BECI, includes each member, each component, main structure, auxiliary structure, and overall technical status evaluation. First of all, the members and components are evaluated; next came the main structure and auxiliary structure; and the overall technical status is the last. If the result of overall technical status is bad enough, the evaluated BECI will be classified as category 4, which implies the replacement should be conducted at once. Otherwise, traditional maintenance will be performed instead.

The evaluation criteria (as shown in Table 1) based on relevant specifications and how bridge inspectors currently evaluate on-site inspections are used for the classification of the technical status of the BECI [29]. Referring to the standards of the transportation industry of the People’s Republic of China, this part introduces the main structure and auxiliary structure of different types of BECIs in detail and serves as the standard for evaluating the technical conditions (as shown in Table 2).

The technical status scores of the members of BECI are calculated as follows:

(1) $$\begin{array}{l} {\mathrm{MMCI}}_{i-l}({\mathrm{AMCI}}_{i-l})=100-\sum_{e=1}^{k}N_{e}\\ \begin{array}{ccc} & N_{e}={\mathrm{DP}}_{ij},e=1 & \end{array}\\ \begin{array}{cc} & N_{e}=\frac{{\mathrm{DP}}_{ij}}{100\times \sqrt{e}}(100-\sum_{r=1}^{e-1}N_{r}) \end{array}\begin{array}{cc} (\mathrm{where} & i=e \end{array}),e\geq 2 \end{array}$$

where ${\mathrm{MMCI}}_{i-l}$ and ${\mathrm{AMCI}}_{i-l}$ are the scores of member l of component *i* for the main structure and auxiliary structure ranging from 0 to 100, which is 0 when ${\mathrm{DP}}_{ij}=100$; *k* is the amount of indicators for the deduction of member l in component *i*; *N*, *e*, and r are referred to as the introduced variables; *i* means the component category, such as side longitudinal girder, rubber sealing belts, or anchorage concrete; *j* means the inspection index *j* of member l of component *i*; ${\mathrm{DP}}_{ij}$ is the deduction value of the inspection index *j* of member l in component *i*, which is calculated according to the inspection index of each member of the component. The deduction value is determined according to Table 3.

The technical status scores of the components of the BECI are calculated as follows:

(2) $${\mathrm{MCCI}}_{i}=\bar{\mathrm{MMCI}}-(100-{\mathrm{MMCI}}_{\min})/t$$

or

(3) $${\mathrm{ACCI}}_{i}=\bar{\mathrm{AMCI}}-(100-{\mathrm{AMCI}}_{\min})/t$$

where ${\mathrm{MCCI}}_{i}$ and ${\mathrm{ACCI}}_{i}$ are the score of component *i* of the main structure and auxiliary structure ranging from 0 to 100, respectively; ${\bar{\mathrm{MMCI}}}_{i}$ and $\bar{{\mathrm{ACCI}}_{i}}$ are the average scores of each member of component *i* of the main structure and auxiliary structure ranging from 0 to 100 respectively; ${\mathrm{MMCI}}_{\min}$ and ${\mathrm{AMCI}}_{\min}$ are the scores of the members with the lowest score in the component i of the main structure and auxiliary structure respectively; *t*, as Table 4 illustrates, is the coefficient that varies with the number of members. 

The technical status scores of main and auxiliary structures of BECI are calculated as follows:

(4) $$\mathrm{SMCI}(\mathrm{SACI})=\overset{m}{\underset{i=1}{\Sigma }}{\mathrm{MCCI}}_{i}({\mathrm{ACCI}}_{i})\times W_{i}$$

where SMCI and SACI are the technical status scores of main and auxiliary structures of the BECI, respectively, whose value ranges from 0 to 100 points; m is the number of types of main structure or auxiliary structure of the BECI; $W_{i}$ is the weight of component *i* which shall be valued in accordance with the recommended values in the standards for technical condition evaluation of highway bridges [32] and AHP (1–9 scale) [33], and the calculated results of different installation are shown in Table 5.

The overall technical status score of BECI is calculated as follows:

(5) $$S_{r}=\mathrm{SMCI}\times W_{\mathrm{SM}}+\mathrm{SACI}\times W_{\mathrm{SA}}$$

where $S_{r}$ is the overall technical status score of BECI, the value range is 0 to 100 points; $W_{\mathrm{SM}}$ and $W_{\mathrm{SA}}$ are the weights of the main structure and auxiliary structure, respectively, which can be valued according to Table 6.

The classification limit of the technical status of the BECI should be implemented according to the regulations in Table 7.

Compared with traditional inspection, this quantitative assessment approach allows an exact number to be outputted when on-site inspectors find the BECI is between two categories and it is difficult to completely make a judgment, which proves the most significant improvement. Additionally, it is worth mentioning that if the inspection index evaluation satisfies the single control index of category 4, it will be classified into category 4 directly without following up the evaluation process. 

Single control indices for BECIs in category 4 are shown as follows:

1. There are more than two serious fractures on the side longitudinal girder of modular BECI.
2. More than 30% of the plate area of the comb plate BECI gets sunk and more than three comb blocks occur.
3. The damage area of elastic expansion body of seamless BECI exceeds 30% of the total area.
4. More than 30% plate area of rubber plate BECI are damaged and fall off.

### 3.2. Selection of Replacement Method Based on AHP

The above replacement plan decisions can be used to determine whether the BECI needs to be replaced, by which the technical status of the BECI is evaluated accurately. According to the whole process of the replacement decision system mentioned above, the performance evaluation should be carried out when the candidate BECI replacement approaches are selected. Then, combined with analytic hierarchy process (AHP), the optimal replacement method based on installation performance is obtained.

#### 3.2.1. Analytic Hierarchy Process

**Figure 3:**
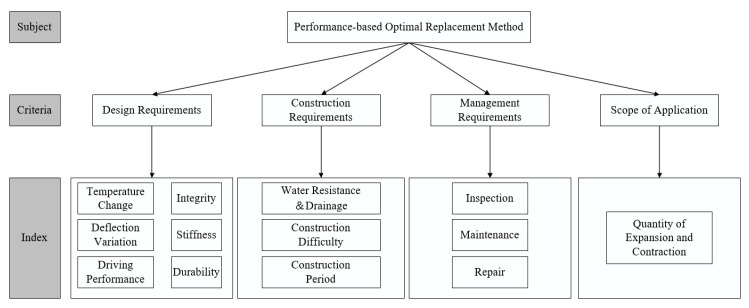
Hierarchy structure model of the performance-based replacement method selection.

The analytic hierarchy process (AHP) is a weight method to determine the indices by multivariate hierarchical processing, which is a method for qualitative analysis of multi-objective objects, sorting the order of good and bad decisions with people’s judgment by representing a complex problem as an order hierarchy. It combines quantitative and qualitative analysis, which is a mathematical thinking process of complex evaluation systems. As is shown in Figure 3, in order to derive the weight of different indices, it usually classifies the evaluation subject into several elements and several hierarchies through complex problems, simple comparison, calculation and judgment between the elements of the same level. Then it will be a reasonable decision basis for selecting the best solution. Design and selection for BECI replacement methods are divided into two major factors: the function coefficient and the cost coefficient. The function coefficient includes four criteria areas: design requirements, construction requirements, management requirement, and scope of application. Every criteria area is further divided into several index areas, including 12 functional indices, which form three levels of the top-to-bottom. As shown in Table 8, most functional indices are qualitative, which can be scored according to the listed evaluation criteria: when the score is higher than 60, it will be qualified, otherwise it will be unqualified.

However, the 12 functional indices are largely qualitative, which it is difficult to describe quantitatively. The reason why their importance requires assessment in an appropriate way is that they are related and need to be evaluated and given a weight. As a complex system, there are many functional indices in the BECI, and most of them cannot be expressed in mathematical terms by quantitative methods. It relies more on an experienced team of experts to evaluate the selection of the BECI by their judgments. If it is not simplified and analyzed, even a team of experienced experts will be unable to judge. In order to facilitate the evaluation, AHP is applied according to the basic design principles of the BECI and various criteria and functional indices are layered to establish a relationship model at each level, then the complex problems will be simplified.

#### 3.2.2. Determination of the Function Coefficients

There are five steps in the realization in the selection and construction of BECI based on AHP, and it is of most importance to determine the weight of criteria area and index area.

##### Establish a Hierarchical Structure Model

The problem of selection and construction of BECI need to be modeled as a hierarchy containing decision subject, criteria for evaluating the alternatives and the indices for reaching it. The selection of BECI will be divided into three levels: subject layer, criteria layer, index layer, which is shown in Figure 3. The factors among the same level do not blend with each other, and that the indices of the previous level have a dominant role in all the indices of the next level.

##### Construct the Judgment Matrix

For the establishment of the priorities among the elements of the hierarchy, a series of judgments should be made based on pairwise comparisons of the elements. After the evaluation of expert team, five judgment matrices A, $B_{1}$, $B_{2}$, $B_{3}$, and $B_{4}$ be formed based on a 1–9 scale method.

The judgment matrix A is listed below:

(6) $$A={\left(a_{ij}\right)}_{4\times 4}=\left\{\begin{array}{cccc} 1 & B_{1}/B_{2} & B_{1}/B_{3} & B_{1}/B_{4}\\ B_{2}/B_{1} & 1 & B_{2}/B_{3} & B_{2}/B_{4}\\ B_{3}/B_{1} & B_{3}/B_{2} & 1 & B_{3}/B_{4}\\ B_{4}/B_{1} & B_{4}/B_{2} & B_{4}/B_{3} & 1 \end{array}\right\}$$

where $B{}_{1}/B_{2}$ is the relative importance of factor $B_{1}$ with respect to factor $B_{2}$, whose specific value is determined in accordance with the result of expert evaluation. In particular, for the subject A, there are four factors $B_{1}$, $B_{2}$, $B_{3}$, $B_{4}$ in the next level which is dominated by A. Then judgments should be carried out according to the 1–9 scale method to determine which factor is most important for A. Hence, if $B_{i}$ is as important as $B_{j}$, the scale of $B_{i}/B_{j}$ is represented by 1; if $B_{i}$ is slightly more important than $B_{j}$, the scale of $B_{i}/B_{j}$ is represented by 3. Accordingly, if $B_{i}$ is more, significantly more, or far more important than $B_{j}$, the scale will be represented by 5, 7, or 9. The intermediate values of the above scales will be 2, 4, 6, and 8 (the influence of the *i*-th factor relative to the *j*-th factor is between the next two adjacent levels). Through the scale of the above numerical values, the judgment matrix can be constructed.

##### Single Hierarchical Arrangement

The normalized eigenvectors ζ which represent the vector of hierarchical weight of the corresponding matrices can be calculated using the square root method as follows [34]:

(7) $$M_{i}=\prod_{j=1}^{n}a_{ij},\bar{\zeta_{i}}=\sqrt[{n}]{M_{i}},\zeta_{i}=\frac{\bar{\zeta_{i}}}{\sum_{j=1}^{n}\bar{\zeta_{j}}}\;(i=1,2,\cdots n)$$

Simultaneously, related maximum eigenvalue is $\lambda_{max}=\sum_{i=1}^{n}\frac{{\left(A\zeta \right)}_{i}}{n\zeta_{i}}$, where ${\left(A\zeta \right)}_{i}$ is the *i*-th part of Aζ.

##### Check the Consistency of Judgment Matrices

The maximum eigenvalues $\lambda_{max}$ acquired above are capable to be applied in the process of checking the consistency of judgment matrices. The relationship between consistency ratio (*CR*) and consistency indicator (*CI*) can be described as follows:

(8) $$\begin{array}{l} CR=\frac{CI}{RI}\\ CI=\frac{\lambda_{max}-n}{n-1} \end{array}$$

where n is the order of matrix; RI is the average random indicator whose value is related to the order of judgment matrix. The numbers of orders 1 to 6 corresponds to 0.00, 0.00, 0.58, 0.90, 1.12, and 1.24, respectively. The closer CR is to zero, the more consistent the judgment matrices prove. Critically, if CR is less than 0.10, the consistency of judgment matrix proves approved and the hierarchical weight is acceptable. Otherwise, A is inconsistent.

##### Determination of the Final Function Coefficients

Based on the hierarchical model above and the result of expert scoring method, the final function coefficient can be determined as follows:

(9) $$D=(B_{1}\cdot \sum_{i=1}^{6}C_{i}\cdot E_{i}+B_{2}\cdot \sum_{i=7}^{9}C_{i}\cdot E_{i}+B_{3}\cdot \sum_{i=10}^{12}C_{i}\cdot E_{i}+B_{4}\cdot E_{4})/100$$

where D is the function coefficient; $B_{i}$ and $C_{i}$ is the hierarchical weight of the criteria and index layers, respectively; $E_{i}$ is the score of factor according to the expert scoring results.

After the process above, the performance-based optimal replacement method is selected via AHP.

### 3.3. Economic Evaluation of the Replacement Method

The cost coefficient—not only economic factors, but also the inspection, monitoring, maintenance cost, and traffic delay—should be taken into account. Normally, the whole life-cycle cost of BECI is comprised of the initial cost of replacement, inspection and maintenance cost, vehicle operating cost, traffic delay cost, accident cost, and life-cycle maintenance cost, etc. However, the environmental protection cost due to BECI construction is not considered due to its complexity and variability.

#### 3.3.1. Initial Cost $CT_{1}$

The initial replacement cost of BECI includes labor, expansion and contraction installation purchase cost, management fee, and other related costs. For example, the replacement cost of a steel comb-type BECI with a displacement box (the amount is 160 mm) is about 7000 CNY/m, which is about 6000 CNY/m for the modular BECI.

#### 3.3.2. Inspection and Maintenance Cost $CT_{2}$

Daily inspections and maintenance are considered small-scale maintenance work. Normally, it will not affect the capacity of the bridge. The cost involved only includes labor costs, which can be determined by regression analysis of the local multi-year data. In the life-cycle, this cost does not change, so the cost of regular maintenance and routine inspection of the life-cycle can be calculated as follows:

(10) $$CT_{2}=C_{a}[\frac{{\left(1+i\right)}^{N}-1}{i{\left(1+i\right)}^{N}}]$$

where $C_{a}$ is the cost of annual maintenance and inspection (CNY); *i* is the social discount rate based on the bank interest rate (%); *N* is the life-cycle of BECI.

#### 3.3.3. Vehicle Operating Cost $CT_{3}$

Vehicles will pass the construction or maintenance zone of the bridge slowly, which will increase the operating cost of vehicles. If the maintenance work of the bridge does not lead to the closure of the road, the vehicle can pass slowly, and the operating cost of the vehicle is calculated as:

(11) $$CT_{3}=(\frac{L}{S_{a}}-\frac{L}{S_{n}})\times \;ADT\times N\times r\;$$

where L is length of road affected; $S_{a}$ is the vehicle speed during maintenance of the bridge; $S_{n}$ is the normal vehicle speed; ADT is the average daily traffic flow; N is the number of days of maintenance; r is the weighted average of vehicle costs.

If the bridge maintenance work leads to the closure of road, vehicles must detour, and the cost of the vehicle operation is calculated as:

(12) $$CT_{3}=(\frac{L_{1}}{S_{n}}-\frac{L}{S_{a}})\times \;ADT\times N\times r\;$$

where $L_{1}$ is the length of detour.

#### 3.3.4. Traffic Delay Cost $CT_{4}$

If the maintenance work of BECI is only partially restricted and does not lead to the closure of the road, vehicles can still pass slowly. Then the delay cost is calculated as:

(13) $$CT_{4}=(\frac{L}{S_{a}}-\frac{L}{S_{n}})\times \;ADT\times N\times W\;$$

where *W* is the time cost of the driver per hour.

It is also possible to estimate the cost due to the road closure during maintenance or replacement. If the road is closed and the vehicle must detour, then the cost of the delay of the vehicle is:

(14) $$CT_{4}=(\frac{L_{1}}{S_{a}}-\frac{L}{S_{n}})\times \;ADT\times N\times W\;$$

#### 3.3.5. Accident Cost $CT_{5}$

Due to the maintenance or replacement of BECI, the number of lanes will be reduced and the vehicle speed will slow down accordingly, which may cause traffic accidents. According to the China traffic accident and related data statistics bulletin of 2014, a total of 196,812 traffic accidents occurred with direct property losses of 107.543 million CNY, which implies that the property losses for each traffic accident was about 5500 CNY. Here, only property cost is considered. The accident cost can be calculated by:

(15) $$CT_{5}=L\times \;ADT\times N\times (A_{a}-A_{n})\times C_{a}$$

where $A_{a}$ is the accident rate during construction (times/100 million kilometers); $A_{n}$ is the normal accident rate; C is the cost per traffic accident in CNY/time.

#### 3.3.6. Life-Cycle Maintenance Cost $CT_{6}$

The service life of BECI is assumed to be 20 years, and it will be repaired three times during the service life. For comb plate expansion joint device, the cost is about 5000 (10th year), 10,000 (15th year), and 20,000 (20th year)—35,000 CNY in total. As for the modular expansion joint device, the cost is about 10,000 (10th year), 15,000 (15th year), and 25,000 (20th year)—50,000 CNY in total. 

Consequently, the cost coefficient C can be calculated as follows:

(16) $$C_{x}=\frac{{\left(\sum_{i=1}^{6}CT_{i}\right)}_{x}}{{\sum_{x=1}^{n}\left(\sum_{i=1}^{6}CT_{i}\right)}_{x}}$$

where x is alternative x and n is the number of alternatives.

## 4. Case Study

There are two main types of BECIs that are applied in the national and provincial highways of Fuyang City, Anhui Province, China: the comb plate-type and modular-type, while other types are rare. The BECI of the Jihe Bridge is damaged severely, which is proposed as a case study in this article and needs to be repaired or replaced. 

**Figure 4:**
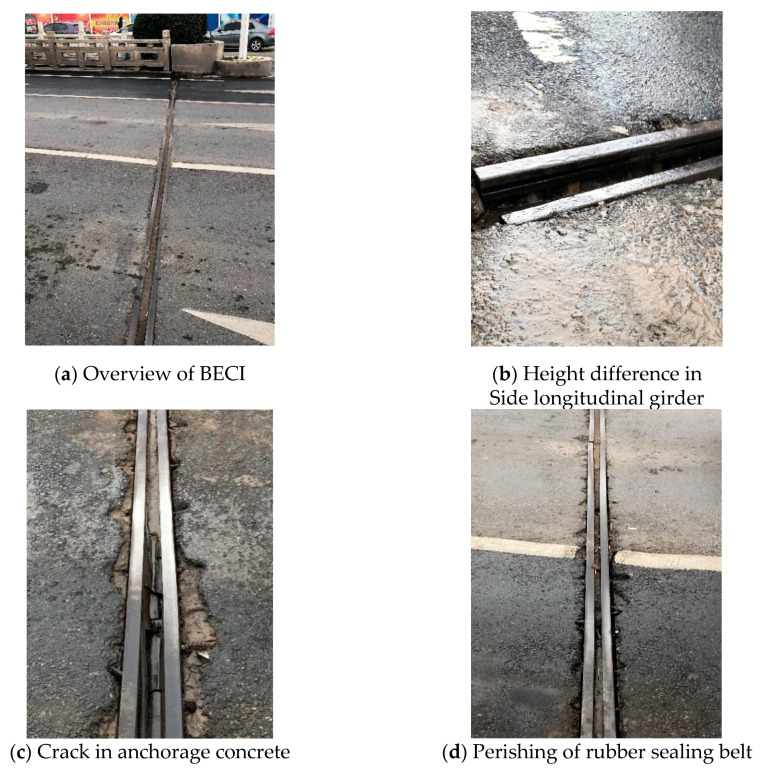
BECI of Jihe Bridge.

Jihe Bridge is located on Beijing East Road, Yingdong District, Fuyang City, with a total length of 24.4 m, a width of 29.4 m and a quantity of expansion and contraction of about 160 mm. In February 2010, the installations were constructed and put into use. After almost 10 years of service life, due to the structural problems of the BECI and the very high traffic volume, enormous amounts of overloaded vehicles, and other factors, the BECIs of the bridge were damaged severely in February 2019 during an on-site inspection, which is shown in Figure 4.

### 4.1. Decisions of Replacement Plan

The photo data of the on-site inspection—brief, yet illuminating—illustrates that the type of BECI used in the Jihe Bridge belongs to the modular MA-type whose overall damage is severe: the side longitudinal girder providing carrying capacity for the vehicles was broken, leading to a serious vehicle bump, and the separation of the anchorage zone and side longitudinal girder occurs, which results in numerous cracks in the anchorage concrete, with the rubber sealing belt mostly falling off. According to the evaluation method, it is evaluated below.

Firstly, the main structure and auxiliary structure of the BECI are divided according to Table 1, and the disease category of each part is preliminarily determined by referring to the evaluation standard: The welding part of the left-side longitudinal girder is broken and this side sinks where the height difference is sizable, causing an obvious vehicle bump phenomenon to occur. According to the evaluation standard, it is seriously damaged and the disease category is 4, while the right side is moderately damaged. There remains an immense number of cracks between both the left and right areas between the anchorage concrete and side longitudinal girder, which results in a slight vehicle bump. According to the evaluation standard, it reflects a medium level of damage, and is classified as category 3; many parts of the rubber sealing belt are seriously aging, cracking, tearing, and leaking where the damaged area exceeds 30% of the total area. According to the evaluation standard, the rubber sealing belt is seriously damaged, and the disease category is 4.

Then, the deduction value of each grade in Table 3 is brought into the comprehensive evaluation of the members. There is only one inspection index in this case, which means the value of k is 1, hence the deduction values ${\mathrm{DP}}_{ij}$ of each member, such as the left- and right-side longitudinal girders, the left and right area of the anchorage concrete, and the rubber sealing belt are 100, 35, 70, 70, and 100, respectively. Then the corresponding technical status scores of each member of BECI are calculated as follows:

$$\begin{array}{l} {\mathrm{MMCI}}_{1-1}=0,\\ {\mathrm{MMCI}}_{1-2}=100-35=65,\\ {\mathrm{MMCI}}_{2-1}={\mathrm{MMCI}}_{2-2}=100-70=30,\\ {\mathrm{BMCI}}_{1}=0 \end{array}$$

In accordance with the Table 4, the values of factor t of the side longitudinal girder, anchorage concrete, and rubber sealing belt are 10, 10, and ∞, respectively. Then, based on the comprehensive evaluation of the components, the above-mentioned evaluation scores are brought in to calculate the scores of each component of the main structure and auxiliary structure, respectively.

$$\begin{array}{l} {\mathrm{MCCI}}_{1}=(65+0)/2-(100-0)/10=22.5,\\ {\mathrm{MCCI}}_{2}=(30+30)/2-(100-30)/10=23,\\ {\mathrm{ACCI}}_{1}=0 \end{array}$$

In this case, referring to Table 5, the weights $W_{i}$ of the side longitudinal girder, anchorage concrete, and rubber sealing belt are 0.6, 0.4, and 1, respectively. Then, the score values of the above-mentioned components are brought in to calculate the score values of the technical condition of the main structure and auxiliary structure of the BECI:

SMCI=22.5×0.6+23×0.4=22.7, SACI=0×1=0

According to the comprehensive evaluation of the main and auxiliary structures, the weight of the main structure is 0.8, and the weight of the auxiliary structure is 0.2. The score value of the above-mentioned structures is brought in to calculate the comprehensive evaluation result of the overall technical status of the BECI as follows:

$$S_{r}=22.7\times 0.8+0\times 0.2=18.16$$

According to $S_{r}=18.16$ and Table 7, it can be determined that the technical condition of the BECI is classified as category 4, which has been completely damaged. It is recommended to replace it for maintenance.

### 4.2. Selection of Replacement Method Based on AHP

The evaluation phase consists of three steps: calculation of the function coefficients, calculation of the cost coefficients, and analysis of the values. The three steps led to a selection of the best alternative, and the selection process is detailed in this section. Based on VE theory, values from each alternative are measured based on the level of function coefficients and the cost coefficients relative to the different selection. The goal is to select the alternative that would offer the largest value.

The following two selection schemes are investigated in the paper.

(1) Alternative A: Comb plate BECI (the expansion and contraction amount is 40–1000 mm or more);
(2) Alternative B: Modular BECI (the expansion and contraction amount can be 80 mm for one unit and up to 2000 mm in combination).

From the main functional advantages and deficiencies of the two types of BECI, it can be concluded that, for criterion $B_{1}$ of the comb plate BECI (design requirement), the adoption to temperature change and deflection variation and driving performance of alternative A are better than those of alternative B, which are the inverse for integrity and stiffness, and there are no major differences on durability. For criterion $B_{2}$, the water resistance and drainage performance of alternative B is better than that of alternative A; during the construction phase, the difficulty in construction of alternative A is smaller than that of alternative B, and the construction period is expected to be shorter than alternative B. Additionally, for alternative A, it is more convenient in inspection, maintenance, and repair, etc.

Based on the 1–9 scale method, and in accordance with the result of expert scoring, the judgment matrices are established as follows:

$$A=\left\{\begin{array}{cccc} 1 & 1 & 2 & 3\\ 1 & 1 & 2 & 3\\ 1/2 & 1/2 & 1 & 2\\ 1/3 & 1/3 & 1/2 & 1 \end{array}\right\},\;B_{1}=\left\{\begin{array}{cccccc} 1 & 6 & 6 & 4 & 4 & 3\\ 1/6 & 1 & 1 & 1/3 & 1/3 & 1/4\\ 1/6 & 1 & 1 & 1/3 & 1/3 & 1/4\\ 1/4 & 3 & 3 & 1 & 1 & 1/2\\ 1/4 & 3 & 3 & 1 & 1 & 1/2\\ 1/3 & 4 & 4 & 2 & 2 & 1 \end{array}\right\},\;B_{2}=\left\{\begin{array}{ccc} 1 & 1/3 & 2\\ 3 & 1 & 4\\ 1/2 & 1/4 & 1 \end{array}\right\},\;B_{3}=\left\{\begin{array}{ccc} 1 & 1/2 & 1\\ 2 & 1 & 2\\ 1 & 1/2 & 1 \end{array}\right\}$$

The results of the eigenvector and maximum eigenvalue calculations via the square root method are shown below:

$$\zeta_{A}={\left(0.35,0.35,0.19,0.11\right)}^{T},\;\zeta_{B1}={\left(0.43,0.05,0.05,0.13,0.13,0.21\right)}^{T},\;\zeta_{B2}={\left(0.24,0.63,0.14\right)}^{T},$$

$$\zeta_{B3}={\left(0.25,0.50,0.25\right)}^{T}\;\mathrm{and}\;\lambda_{maxA}=4.02,\;\lambda_{maxB1}=6.13,\;\lambda_{maxB2}=3.06,\;\lambda_{maxB3}=3.00.$$

Next comes to the process of consistency test:

$$\mathrm{Matrix}\;A:\;CI=0.007,\;RI=\;0.9,\;CR=0.007<0.1;\;\mathrm{Matrix}\;B_{1}:\;CI=\;0.026,\;RI=1.24,\;CR=0.02<0.1.$$

$$\mathrm{Matrix}\;B_{2}:\;CI=0.03,\;RI=0.58,\;CR=0.05<0.1;\;\mathrm{Matrix}\;B_{3}:\;CI=0.00,\;RI=0.58,\;CR=0.00<0.1.$$

All the values of CR are within the allowable range of 0.10, which prove the consistency of the judgment matrices are acceptable. Consequently, all the factors mentioned above, including the final function coefficients, are collected in Table 9. 

### 4.3. Economic Evaluation of the Replacement Method

The life-cycle cost of BECI can be calculated according to the parameters shown in Table 10. In this step of the analysis, only the initial cost, annual routine maintenance, and inspection fee, as well as the three main repair costs, are considered for the selection of the BECI. Therefore, the cost coefficients of the two schemes are 0.49 and 0.51 for alternative A and B, respectively.

The last step in determining the value coefficient for the two alternatives is to calculate the value based on the value engineering. The decision rule is that when the value of one alternative significantly outweighs that of the other, it implies that the alternative has a greater opportunity for selection. Therefore, based on the function and cost coefficient from Table 9 and Table 10, the results show that the function coefficient, cost coefficient and value coefficient (the ratio of the function coefficient and cost coefficient) of alternative A are 0.857, 0.49, and 1.75, respectively, and for alternative B are 0.841, 0.51, and 1.65, respectively. It can be seen that the coefficients of the two alternatives are both greater than 1.00, which implies that the two alternatives are both reasonable, and the value coefficient of alternative A is larger than that of alternative B; subsequently, alternative A is preferential for application of this project.

## 5. Summary and Conclusions

A quantitative assessment approach for selecting a BECI replacement method is proposed, and a replacement method decision system based on installation performance and economic evaluation is constructed. In the first stage of the decision process, the BECI overall technical status is evaluated, which provides a strong basis for determining "whether or not to replace". In the second stage, AHP is fully utilized in the installation performance evaluation to select a performance-based optimal replacement method. In the third stage, a calculation model based on LCVA is established for economic evaluation to satisfy the economic requirement. Eventually, according to the results gained above, a comprehensive evaluation is conducted to rank the values of the candidate replacement methods and select the optimal one. The case study illustrates that the constructed BECI replacement decision system and related optimization methods developed by this research are feasible and efficient. The decision system proves a reliable DM tool in bridge O and M.

Based on the findings, the optimal replacement method with both higher function coefficient and lower economic coefficient can not only fulfil the performance requirements but also pursue a cost reduction, which leads to a considerable value increment. Additionally, both managers and front-line staff are entitled to directly participate in DM. Compared with traditional inspection, the quantitative assessment approach proposed allows an exact number to be outputted when on-site inspectors find the BECI is between two categories and it is difficult to completely make a judgment, which proves the most significant improvement. In conclusion, the systematic research not only standardizes the process of assessment, but also emphasizes the centrality of people themselves. The efficiency improvement and cost reduction are pursued for those who are the DM subjects themselves, hence every operator in the system has the opportunity to be empowered and motivated to make decisions.

Especially, in order to refine and simplify the quantitative assessment approach, this study does not take the scope of the adaptation of the BECI, which is generally referred to as the quantity of expansion and contraction into account when carrying out an installation performance evaluation. In actual application, the decision-makers should preselect the candidate replacement methods according to the scope of the adaptation of the BECI, so as to ensure the integrity and efficiency of the decision system. Further research should also be conducted on the impacts of the scope of adaptation on installation performance, which could improve the constructed system.

## Figures and Tables

**Table 1:** Technical status assessment categories.

Category	Technical Status Description of BECI	Technical Status Description of Main or Auxiliary Structure
1	Brand new state, perfect function.	Brand new state, perfect function.
2	The function is good, slight defects in the main structure and auxiliary structure which can be eliminated by strengthening inspections and normal maintenance occurs without affecting normal use.	The function is good, slight defects in materials and components which can be eliminated by strengthening inspection and normal maintenance occur without affecting normal use.
3	The function is reduced, and the main structure and auxiliary structure are partially damaged which can be used normally through maintenance and component replacement.	The function is reduced, and the materials and components are partially damaged which can be used normally through maintenance and component replacement.
4	Serious functional diseases occur, the main structure and auxiliary structure are severely defective and cannot be used, seriously affecting the safety of driving.	Serious functional diseases occur, the materials and components are severely defective and cannot be used, seriously affecting the safety of driving.

**Table 2:** Main structure and auxiliary structure of the BECI.

No.	Installation	Main Structure	Auxiliary Structure	Criterion
1	Modular (MA type)	Side longitudinal girder, anchorage concrete	Rubber sealing belt	JT/T 327 [1]
	Modular (MB type)	Side longitudinal girder, medium longitudinal girder, anchorage concrete, transverse beam, displacement box, bearing	Rubber sealing belt	
2	Comb plate (SC type)	Comb plate, stainless steel plate, anchorage concrete	Water guiding device	
	Comb plate (SSA type)	Fixed comb plate, movable comb plate, stainless steel plate, anchor bolt, displacement box, anchorage concrete	Water guiding device	JT/T 327, JT/T 723 [30]
	Comb plate (SSB type)	Fixed comb plate, movable comb plate, stainless steel plate, anchor bolt, displacement box, anchorage concrete	Water guiding device	
3	Seamless type	Elastic expansion body, steel plate, nail	Isolating membrane	
4	Wave-type	Corrugated plate, u-shaped groove, foam stick	Special sealant	JT/T 502 [31]
5	Rubber plate type	Rubber plate, stiffened steel plate, anchor bolt	Sealing strip	JT/T 327

**Table 3:** Deduction values of each inspection index of the member.

The Highest-Level Category that Can Be Achieved by Inspection Indices	Index Category			
	1	2	3	4
3	0	25	50	/
4	0	35	70	100

**Table 4:** Value of “t” factor.

n	t	n	t
1	∞	20	6.6
2	10	21	6.48
3	9.7	22	6.36
4	9.5	23	6.24
5	9.2	24	6.12
6	8.9	25	2.00
7	8.7	26	5.88
8	8.5	27	5.76
9	8.3	28	5.64
10	8.1	29	5.52
11	7.9	30	5.4
12	7.7	40	4.9
13	7.5	50	4.4
14	7.3	60	4.0
15	7.2	70	3.6
16	7.08	80	3.2
17	6.96	90	2.8
18	6.84	100	2.5
19	6.72	≥200	2.3

Note: n is the total number of members of component i; The t values not listed in the table are calculated by interpolation.

**Table 5:** Weight of the component for BECI.

Installation	Part	Category	Component	$W_{i}$
Modular (MA type)	Main structure	1	Side longitudinal girder	0.6
		2	Anchorage concrete	0.4
	Auxiliary structure	3	Rubber sealing belt	1
Modular (MB type)	Main structure	1	Side longitudinal girder	0.1
		2	Medium longitudinal girder	0.3
		3	Transverse beam	0.08
		4	Displacement box	0.12
		5	Bearing	0.2
		6	Anchorage concrete	0.2
	Auxiliary structure	7	Rubber sealing belt	1
Comb plate (SC)	Main structure	1	Comb plate	0.4
		2	Stainless steel plate	0.5
		3	Anchorage concrete	0.1
	Auxiliary structure	4	Water guiding device	1
Comb plate (SSA)	Main structure	1	Fixed comb plate	0.15
		2	Movable comb plate	0.35
		3	Stainless steel plate	0.05
		4	Anchor bolt	0.1
		5	Displacement box	0.05
		6	Anchorage concrete	0.3
	Auxiliary structure	7	Water guiding device	1
Comb plate (SSB)	Main structure	1	Fixed comb plate	0.15
		2	Movable comb plate	0.35
		3	Stainless steel plate	0.05
		4	Anchor bolt	0.1
		5	Displacement box	0.05
		6	Anchorage concrete	0.3
	Auxiliary structure	7	Water guiding device	1
Seamless type	Main structure	1	Elastic expansion body	0.8
		2	Steel plate	0.15
		3	Nail	0.05
	Auxiliary structure	4	Isolating membrane	1
Wave-type	Main structure	1	Corrugated plate	0.35
		2	U-shaped groove	0.35
		3	Foam stick	0.3
	Auxiliary structure	4	Special sealant	1
Rubber plate type	Main structure	1	Rubber plate	0.6
		2	stiffened steel plate	0.27
		3	Anchor bolt	0.13
	Auxiliary structure	4	Sealing strip	1

**Table 6:** Weight of main structure and auxiliary structure.

Part	Weight
Main structure	0.8
Auxiliary structure	0.2

**Table 7:** Classification table of technical status of the BECI.

Technical Status Score	$\mathrm{Technical}\;\mathrm{Status}\;S_{j}$			
	Category 1	Category 2	Category 3	Category 4
$S_{r}$ (SMCI, SACI)	[90, 100]	[75, 90)	[50, 75)	[0, 50)

**Table 8:** Evaluation basis of function coefficients.

Criteria		Index	
B1	Design requirements	C1	Temperature change
		C2	Deflection variation
		C3	Driving performance
		C4	Integrity
		C5	Stiffness
		C6	Durability
B2	Construction requirements	C7	Water resistance and drainage
		C8	Construction difficulty
		C9	Construction period
B3	Management requirements	C10	Inspection
		C11	Maintenance
		C12	Repair
B4	Scope of application	/	/

**Table 9:** Values of the function coefficients of alternatives A and B.

Layer	Item	Weight												
Criteria	Criteria	B1						B2			B3			B4
	Weight	0.35						0.35			0.19			0.11
Index	Index	C1	C2	C3	C4	C5	C6	C7	C8	C9	C10	C11	C12	/
	Weight	0.43	0.05	0.05	0.13	0.13	0.21	0.24	0.63	0.14	0.25	0.50	0.25	/
Alternative A	E	85	80	80	90	90	85	80	85	80	90	90	90	85
	D	0.857												
Alternative B	E	90	90	85	85	80	85	90	80	75	80	80	80	90
	D	0.841												

**Table 10:** The parameters of the cost coefficient calculation for alternatives A and B.

Item	Unit	Alternative A	Alternative B
Initial cost ($CT_{1}$)	CNY	7000	6000
Annual routine inspection and maintenance fees ($C_{a}$)	CNY	1000	1000
Social discount rate (*i*)	/	4%	4%
Repair cost	CNY/time	35,000	50,000
Life-cycle of the BECI (n)	year	20	20

## Data Availability

The data used to support the findings of this study are available from the corresponding author upon request.
